# Alcance de Metas de LDL-colesterol: Por que Falhamos e Como Podemos Melhorar?

**DOI:** 10.36660/abc.20220288

**Published:** 2022-06-06

**Authors:** Fernando Cesena

**Affiliations:** 1 Cenocor Guarulhos SP Brasil Cenocor, Guarulhos, SP – Brasil

**Keywords:** Doenças Cardiovasculares/prevenção e controle, Infarto do Miocárdio, Hipercolesterolemia, Agentes Anticolesterolemiantes, Qualidade da Assistência à Saúde, Fatores de Risco

A doença aterosclerótica cardiovascular (DACV) continua sendo a primeira causa de morte no mundo e no Brasil.^1, 2^ Indivíduos com DACV prévia estão em maior risco de eventos subsequentes, e as diretrizes recomendam uma redução agressiva dos níveis de colesterol da lipoproteína de baixa densidade (LDL-c) para evitar desfechos ruins.^3, 4^

No entanto, vários relatos de todo o mundo indicam uma lacuna entre as recomendações das diretrizes e a prática clínica, e grande parte da população, principalmente na prevenção secundária, vive com níveis de LDL-c acima dos considerados razoáveis para prevenir eventos.^5-8^ De fato, a falta de adesão às terapias recomendadas pelas diretrizes foi independentemente associada a eventos cardiovasculares maiores em uma população brasileira após síndrome coronariana aguda.^9^

Nesse contexto, Bernardi et al. relatam os níveis de LDL-c após infarto do miocárdio na cidade de Curitiba-PR, Brasil. Os autores analisaram retrospectivamente pacientes admitidos por infarto do miocárdio em hospitais públicos entre 2008 e 2015. Entre 1.451 pacientes avaliados 33 meses em média após o evento, apenas 29% e 7% apresentaram nível de LDL-c <70 mg/dL e <50 mg /dL, respectivamente, enquanto o LDL-c foi ≥100 mg/dL em 36% da amostra.^10^

Essas valiosas informações lançam luz sobre um antigo debate: por que é tão difícil atingir as metas de LDL-c e como podemos melhorar? A resposta é nada menos que complexa e deve envolver várias partes.

Os médicos podem não conhecer as diretrizes, podem não concordar com elas ou podem temer níveis muito baixos de LDL-c. No entanto, a melhor evidência de ensaios clínicos randomizados apoia não apenas a eficácia, mas também a segurança da redução agressiva do LDL-c em pacientes de alto risco.^4^ Alguns médicos são afetados pela inércia clínica. Outros podem achar que não há diferença substancial entre manter o LDL-c <50, 70 ou 100 mg/dL. Vale lembrar que o impacto das estratégias preventivas na redução do risco absoluto aumenta com o tempo, diminuindo o número necessário para tratar (NNT) para prevenir um evento na perspectiva de longo prazo da DACV.

Por outro lado, os pacientes podem subestimar o risco e desconhecer as metas de LDL-c,^11^ podem superestimar a eficácia de estratégias não farmacológicas e minimizar a necessidade de tratamento medicamentoso, podem não poder pagar os medicamentos ou simplesmente não aderir a eles por vários motivos, incluindo o desenvolvimento de sintomas musculares ou o medo exagerado de efeitos adversos. No entanto, é amplamente aceito que o efeito nocebo é altamente prevalente e uma verdadeira intolerância às estatinas é muito menos comum do que muitos podem pensar.^4^

Se o objetivo final é implementar terapias baseadas em evidências com sucesso, a educação médica continuada e campanhas públicas são essenciais, mas não suficientes. Medidas mais profundas, amplas e impactantes devem ser discutidas. Precisamos levar essa questão mais a sério.

Ações de valorização e resgate do método científico como motor central das decisões médicas seriam bem-vindas, servindo de contraponto às práticas alternativas e à pseudociência que têm conquistado a simpatia de tantas pessoas, inclusive médicos. As escolas médicas e os profissionais de saúde têm papel fundamental nesse processo.

É imperativo identificar corretamente as barreiras à implementação das diretrizes, que podem variar de acordo com a região, ambiente (prática pública versus privada, atenção primária versus especializada) ou condições socioeconômicas. Os fatores identificados devem ser alvos para programas de melhoria da qualidade. No Brasil, há bons exemplos a serem seguidos, como o programa Boas Práticas em Cardiologia adaptado do programa Get With The Guidelines da American Heart Association,^12^ e intervenções de melhoria da qualidade testadas em ensaios randomizados por cluster.^13, 14^

No nível institucional, estabelecer métricas e metas de desempenho, auditorias independentes, programas de acreditação e modelos de pagamento baseados em valor são propostas que podem ser debatidas para melhorar a qualidade da assistência médica. Ao nível do médico, deve ser considerada a avaliação periódica da competência para exercer a Medicina.

As tecnologias modernas precisam ser aproveitadas na busca pela melhoria da qualidade dos cuidados de saúde. É cada vez mais fácil identificar pacientes em risco que não atingem as metas de LDL-c ou não têm os lipídios plasmáticos dosados. Alertas automáticos por meio de telefones celulares ou e-mails incentivando esses indivíduos a procurar atendimento médico podem encontrar um lugar nesse contexto. Além disso, a telemedicina permite a integração entre a atenção básica e os centros especializados e pode ser útil para o manejo de casos mais complexos.

Por fim, todos os esforços mencionados acima são inúteis se o acesso ao tratamento farmacológico adequado permanecer restrito. No Brasil, a maioria dos indivíduos depende do sistema público de saúde e tem acesso apenas às estatinas de menor potência.^15^ Há uma necessidade urgente de facilitar a disponibilidade de atorvastatina, rosuvastatina e ezetimiba, pelo menos para aqueles que precisam delas para atingir as metas de LDL-c.

Em conclusão, o desenvolvimento de diretrizes é inútil se as recomendações não forem aplicadas à população. A implementação das melhores evidências científicas sobre a redução do LDL-c na prática clínica é um desafio. A educação médica e do paciente são os pilares para o sucesso, mas são necessárias atitudes mais abrangentes. Diferentes setores da sociedade, incluindo gestores de saúde, formuladores de políticas, sociedades médicas e conselhos de regulaçao profissional, devem assumir essa responsabilidade.
