# Supplementary material for: Alcance de Metas de LDL-colesterol: Por que Falhamos e Como Podemos Melhorar?
Source: Arq Bras Cardiol. 2022 Jun 6;118(6):1026–7. [Article in Portuguese] doi: 10.36660/abc.20220288 (PMC9345154; doi:10.36660/abc.20220288)
Supplement: Translation

# Translation: Achievement of LDL-cholesterol Targets: Why do We Fail, and How Can We Improve?

**Authors:** Fernando Cesena

**Affiliations:** 1 Guarulhos SP Brazil Cenocor, Guarulhos, SP – Brazil

**Keywords:** Cardiovascular Diseases, Hypercholesterolemia, Anticholesteremic Agents, Practice Guideline, Quality of Health Care

Atherosclerotic cardiovascular disease (ASCVD) remains the first cause of death in the world, and Brazil.^1, 2^ Individuals with previous ASCVD are at the highest risk of subsequent events, and guidelines recommend aggressive lowering of low-density lipoprotein cholesterol (LDL-c) levels to prevent bad outcomes.^3, 4^

However, several reports from around the world indicate a gap between guideline recommendations and clinical practice, and a large proportion of the population, especially in secondary prevention, lives with LDL-c levels above those considered reasonable to prevent events.^5-8^ Indeed, lack of adherence to guideline-recommended therapies was independently associated with major cardiovascular events in a Brazilian population after acute coronary syndrome.^9^

In this context, Bernardi et al. report on LDL-c levels after myocardial infarction in the city of Curitiba-PR, Brazil. The authors retrospectively analyzed patients admitted for myocardial infarction in public hospitals between 2008 and 2015. Among 1451 patients evaluated 33 months on average after the event, only 29% and 7% had an LDL-c level <70 mg/dL and <50 mg/dL, respectively, while LDL-c was ≥100 mg/dL in 36% of the sample.^10^

This valuable information sheds light on an old debate: why is it so hard to achieve LDL-c targets, and how can we improve? The answer is nothing less than complex and should involve multiple parts.

Physicians may not know the guidelines, may not agree with them or may fear too low LDL-c levels. However, the best evidence from randomized clinical trials supports not only the efficacy but also the safety of aggressive LDL-c lowering in high-risk patients.^4^ Some physicians are affected by clinical inertia. Others may feel that there is no substantial difference between keeping LDL-c <50, 70, or 100 mg/dL. It is worth remembering that preventive strategies’ impact on absolute risk reduction increases with time, decreasing the number needed to treat (NNT) to prevent one event in the long-term perspective of ASCVD.

Conversely, patients may underestimate the risk and be unaware of LDL-c targets,^11^ may overestimate the efficacy of non-pharmacological strategies and downplay the need for drug treatment, may not afford the medications, or be just non-adherent to them due to several reasons, including the development of muscle symptoms or exaggerated fear of adverse effects. However, it is widely accepted that the nocebo effect is highly prevalent, and a real statin intolerance is far less common than many can think.^4^

If the final goal is to implement evidence-based therapies successfully, continuing medical education and public campaigns are essential but not enough. Deeper, broader, and more impactful measures should be discussed. We need to take this issue more seriously.

Actions to valorize and rescue the scientific method as the core driver of medical decisions would be welcome, serving as a counterpoint to alternative practices and pseudoscience that have gained the sympathy of so many people, including medical doctors. Medical schools and health professionals have a fundamental role in this process.

It is imperative to correctly identify the barriers to guideline implementation, which may vary according to the region, setting (public versus private practice, primary versus specialized care), or socioeconomic conditions. The identified factors should be targets for quality improvement programs. In Brazil, there are good examples to follow, such as the Best Practice in Cardiology program adapted from the American Heart Association’s Get With The Guidelines Program,^12^ and quality improvement interventions tested in cluster randomized trials.^13, 14^

At the institutional level, establishing performance metrics and goals, independent audits, accreditation programs, and value-based payment models are proposals that can be debated to improve healthcare quality. At the physician level, periodic assessment of competence to practice Medicine should be considered.

Modern technologies need to be leveraged in the quest for improving healthcare quality. It is increasingly easier to identify at-risk patients who do not achieve LDL-c targets or do not have plasma lipids measured. Automatic alerts via mobile phones or e-mails encouraging such individuals to seek medical care may find a place in this context. Moreover, telemedicine allows integration between primary care and expert centers and may be useful for managing more complex cases.

At last, all the efforts mentioned above are worthless if the access to adequate pharmacological treatment remains restricted. In Brazil, most individuals depend on the public health system and have access only to the lowest-potent statins.^15^ There is an urgent need to facilitate the availability of atorvastatin, rosuvastatin, and ezetimibe, at least for those who need them to attain LDL-c targets.

In conclusion, guideline development is useless if the recommendations are not applied to the population. Implementing the best scientific evidence regarding LDL-c lowering in clinical practice is challenging. Medical and patient education are the pillars to succeed, but more comprehensive attitudes are needed. Different sectors of society, including health managers, policymakers, medical societies, and professional regulators, should take this responsibility.
